# Platinum (II) Compounds With Antitumor Activity Studied by Molecular Mechanics

**DOI:** 10.1155/MBD.1998.91

**Published:** 1998

**Authors:** Natasha Trendafilova, Ivelina Georgieva, George St. Nikolov

**Affiliations:** Institute of General and Inorganic Chemistry Bulgarian Academy of Sciences Sofia 1113 Bulgaria

## Abstract

A series of Pt(ll) complexes with antitumor properties: [1,2-bis(2,6-dichloro-4-hydroxyphenyl)ethylenediamine]PtL_2_ (meso-1-PtL_2_) and [erythro-1-(2,6-dichloro-4-hydroxyphenyl)-2-(2-halo-4-hydroxyphenyl)ethylenediamine]PtL_2_, [2L=2Cl−,2I−,SO_4_^2^−; halo = F (erythro-8-PtL_2_),halo = Cl (erythro-9-PtL_2_)] has been modelled by molecular mechanics (MM). The MM calculations were carried out for different isomers and ligand conformations meso-δ, meso-λ, d,l-δ, d,I-λ. The compounds with the lowest MM energies have the same geometries as those obtained by X-ray analysis. The calculated MMX energy orders: meso-1-PtL_2_ < erythro-9-PtL_2_ < erythro-8-PtL_2_ for L=I−, Cl− and SO_4_^2−^ are reverse to the known antitumor activity order - the lowest energy complex (the most stable one)is the one with the highest estrogen activity (meso-1-PtL_2_). The type of the leaving group (L) does not alter the energy order, which is in agreement with the biological experiments that show a slight dependence of the estrogen properties on the leaving group type.


### PLATINUM (II) COMPOUNDS WITH ANTITUMOR ACTIVITY STUDIED BY MOLECULAR MECHANICS

Natasha Trendafilova*, Ivelina Georgieva and George St. Nikolov

Institute of General and Inorganic Chemistry, Bulgarian Academy of Sciences, 1113 Sofia, Bulgaria

### Abstract

A series of Pt(II) complexes with antitumor properties: [1,2-bis(2,6-dichloro-4-hydroxyphenyl)ethylenediamine]$PtL_2$ (meso-1-$PtL_2$) and [erythro-1-(2,6-dichloro-4-hydroxyphenyl)-2-(2-halo-4-hydroxyphenyl)ethylenediamine]$PtL_2$, [2L = 2$Cl^-$, 2$I^-$, $SO_4^{2-}$; halo = F (erythro-8-$PtL_2$), halo = Cl (erythro-9-$PtL_2$)] has been modelled by molecular mechanics (MM). The MM calculations were carried out for different isomers and ligand conformations meso-δ, meso-λ, d,l-δ, d,l-λ. The compounds with the lowest MM energies have the same geometries as those obtained by X-ray analysis. The calculated MMX energy orders: meso-1-$PtL_2$ < erythro-9-$PtL_2$ < erythro-8-$PtL_2$ for L = $I^-$, $Cl^-$ and $SO_4^{2-}$ are reverse to the known antitumor activity order - the lowest energy complex (the most stable one) is the one with the highest estrogen activity (meso-1-$PtL_2$). The type of the leaving group (L) does not alter the energy order, which is in agreement with the biological experiments that show a slight dependence of the estrogen properties on the leaving group type.

### Introduction

The coordination compounds of Pt(II) are among the most important antitumor reagents. Cis-$Pt(NH_3)_2Cl_2$(cisplatin) was the first platinum compound tested on a wide scale for cytostatic effects [1-4]. This compound, however, is toxic and in some cases not selective enough [5]. A new class of Pt(II) compounds, designed by combining the cytotoxic $PtCl_2$ group (the active moiety in cisplatin) and a diamine ligand with estrogen-receptor affinity has been found [6-9]. These new complexes retain the estrogen properties of the ligand that binds to the estrogen receptor which is specific for the tumor cell and thus the complexes attack selectively critical areas of the DNA [9-15]. Such compounds are more selective and less toxic [6]. The synthetic estrogen, hexestrol (HES-nonsteroidal) (Fig. 1) has been used as a model of a ligand with estrogen-receptor affinity [7]. By exchange of the ethyl side chains with amino groups, HES was transformed into a compound suitable for coordination to Pt(II) (Fig. 1). After this structural modification, however, HES loses its high affinity to the estrogen receptor as well as its marked estrogen activity [17]. To increase the lipophilic character of the diamine, two chlorine atoms have been introduced into 2- and 6-positions of the aromatic rings [18]. The compound thus obtained (meso-1) as well as its dichloroplatinum(II) complex (meso-1-$PtCl_2$) proved to be "true" estrogens [9].

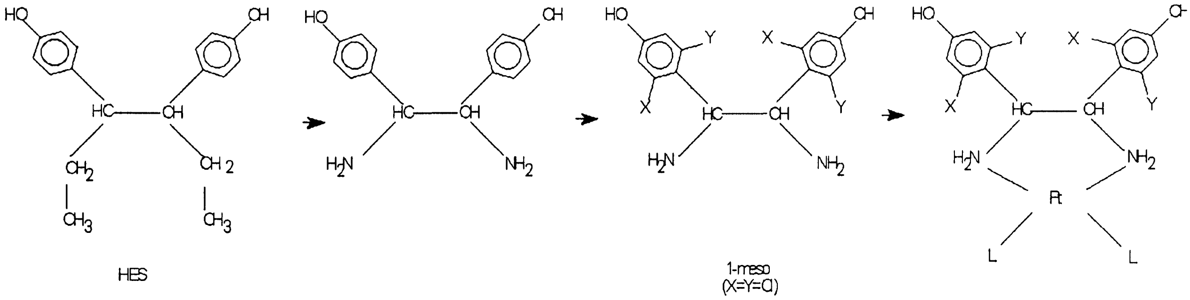

Fig. 1. Transformation of Hexestrol (HES) into a Pt(II) complex with estrogen activity (meso-1-$PtCl_2$) [7].

The compounds [meso-1,2-bis(2,6-dichloro-4-hydroxyphenyl) ethylene-diamine] $PtL_2$ (meso-1-$PtL_2$) and [erythro-1-(2,6-dichloro-4-hydroxyphenyl)-2-(2-halo-4-hydroxyphenyl) ethylenediamine]$PtL_2$ [2L = 2$Cl^-$, 2$I^-$, $SO_4^{2-}$; halo = F (erythro-8-$PtL_2$), halo = Cl (erythro-9-$PtL_2$)] have been found to possess estrogen properties and have been tested as antitumor agents [9,12-22]. The compounds show different estrogen activity [12]. The type (Cl, F, H, OH) and the positions

(*ortho-*, *meta-*, *para-*) of the substituents in the aromatic rings strongly influence their estrogen properties [10,12,15]. At the same time the type of the leaving group ($Cl^-$, $I^-$, $SO_4^{2-}$) was of minor importance for the estrogen activity [18].

The stability of the studied Pt(II) compounds, used as antitumor agents is important for the transport of the complex to the estrogen receptor. The more stable complex will reach the specific estrogen receptor of the tumor cell in higher concentration [23].

Recently, we have showed [24,25] that the thermodynamic stability correlates with the rate of hydrolysis of d,l- and meso-[1,2 - bis (2-hydroxyphenyl) ethylenediamine] dichloroplatinum(II) (3-$PtCl_2$). Among the studied isomers the lowest energy one, d,l-λ shows the highest rate of hydrolysis and highest antitumor activity.

In another theoretical study we have shown that the type and the positions of the ring substituents alter the calculated conformational energies (thermodynamic stabilities) of the studied compounds in agreement with their antitumor activity [26].

The geometry of the active compounds is also important since it will define the bonding mode to the estrogen receptor. Both factors - thermodynamic stability and geometry - cannot be taken from crystal structures since these factors for the compounds in solutions may differ substantially. The molecular structure and thermodynamic stability, however, can readily be approached by molecular modelling.

The purpose of the present work is to examine all possible isomers of the compounds mentioned above, using molecular mechanics. Correlations between the calculated MM energies (thermodynamic stabilities) and the known estrogen activity order are expected to be found. Different leaving groups, ($Cl^-$, $I^-$, $SO_4^{2-}$) were used in the calculations in order to study the influence of the leaving group, L, on the calculated energies and stabilities.

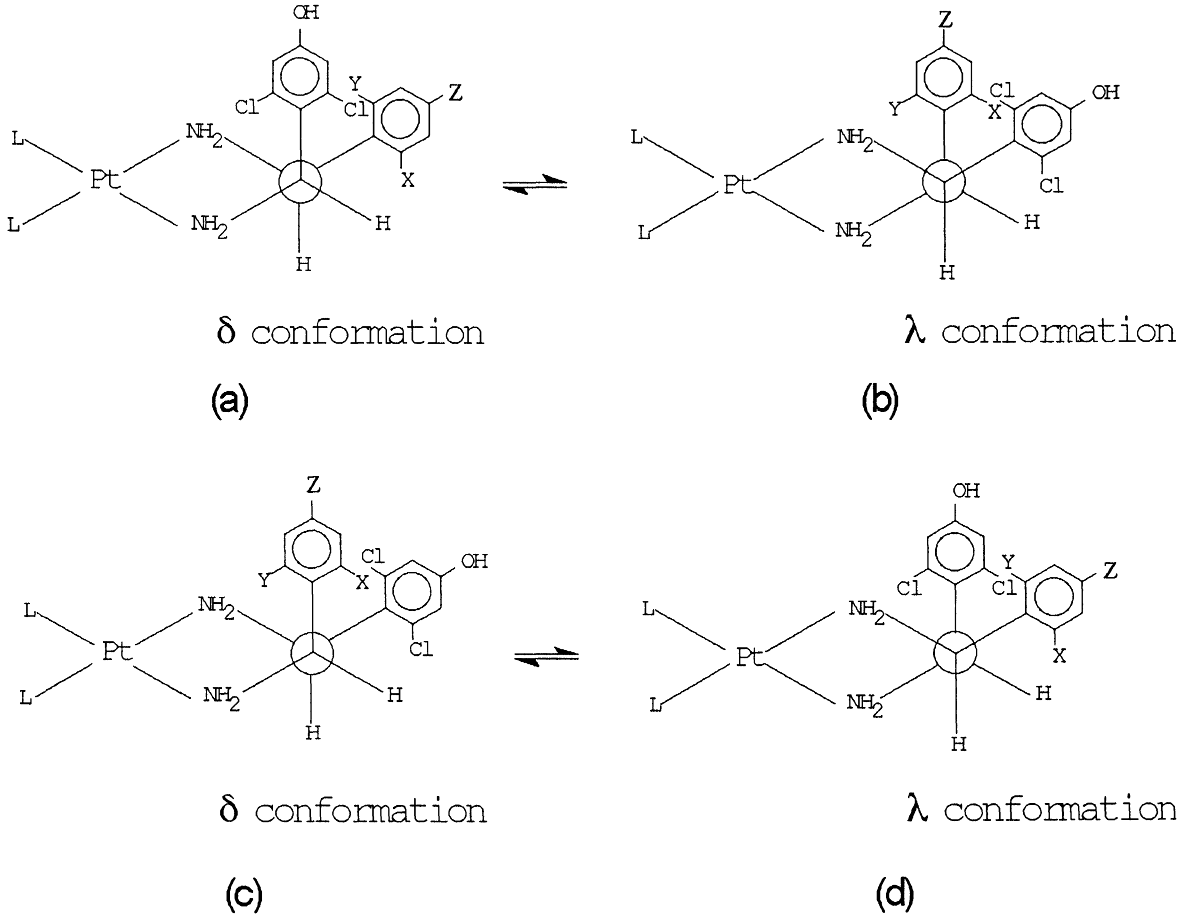

Fig. 2. Different conformations of the meso-1-$PtL_2$ (X=Y=Cl, Z=OH), erythro-5-$PtL_2$ (X=Y=H, Z=OH), erythro-7-$PtL_2$ (X=Y=Cl, Z=H), erythro-8-$PtL_2$ (X=F, Y=H, Z=OH) and erythro-9-$PtL_2$ (X=Cl, Y=H, Z=OH).

**Methods**

Molecular Mechanics is now a well-established technique in the field of inorganic chemistry. It was successfully applied to many coordination compounds to predict and rationalize the conformational behaviour of different metal complexes [27-36]. This approach was successfully applied also for modelling of a number of Pt(II) compound used as anticancer drugs [37].

We have used the standard MMX (an enhanced version of MMP2) procedure with the parameters collected in its 1988 version [38] (see Appendix). The calculated MM energies are used to access the relative stability of the studied complexes as suggested elsewhere [39].

In some cases, namely for cisplatin and its substituted ethylenediamine derivative, MM calculations, which ignore explicitly the electronic factors, gave lower energies for the tetrahedral structures than for the planar ones [24]. To calculate the geometry of the higher energy square-planar structures by the MM method in such cases we fixed the ligand donor atoms in a plane.

**Results and Discussion**

The studied complexes are given in Fig. 2 and Fig. 3:

For the complexes shown in the two Figures the following isomers have been studied:

(i) four meso isomers (one aromatic ring is in axial- and the second in equatorial orientations): among them, two δ conformers, (Fig. 2a and 2c) and two λ conformers (Fig. 2b and 2d);

(ii) one d,l isomer, δ conformer (both aromatic rings are in axial orientations) (Fig. 3a).

(iii) one d,l isomer, λ conformer (both aromatic rings are in equatorial orientations) (Fig. 3b).

We shall now examine the influence of A. - the type of the leaving group (L), B. - ring substituents (X, Y and Z) and C. - rotational barriers about $C_{sp3}$- $C_{ar}$ bond.

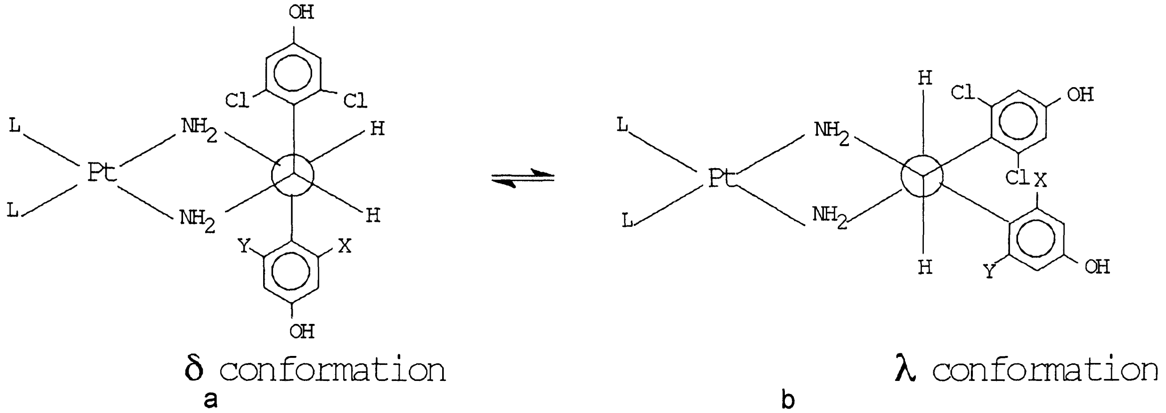

a b

Fig. 3. Different conformations of the d,l-1-$PtL_2$ (X=Y=Cl, Z=OH), threo-5-$PtL_2$ (X=Y=H, Z=OH), threo-7-$PtL_2$ (X=Y=Cl, Z=H), threo-8-$PtL_2$ (X=F, Y=H, Z=OH) and threo-9-$PtL_2$ (X=Cl, Y=H, Z=OH).

**A. Influence of the type of the leaving group on the conformational energies**

The MM energies of the studied compounds were calculated in the presence of three different leaving groups: L = $I^-$, $Cl^-$ and $SO_4^{2-}$. The MM calculations showed that the main contribution to the calculated energy comes from the stretching energy term. Small variations of the Pt-L bond lengths in the $PtI_2$ complexes produce large energy changes. To estimate the correlation E vs. r(Pt-L), the energies associated with bond length distortions were calculated using the Pt-L parameters included in the program (see Appendix). Three meso isomers: meso-1-$PtL_2$, erythro-8-$PtL_2$ and erythro-9-$PtL_2$ with different leaving groups, L = $I^-$, $Cl^-$ and $SO_4^{2-}$ were selected. These three compounds were tested and they showed estrogen affinity and activity [18]. The curves obtained are given in Fig. 4. For the studied compounds the energy minima were found at Pt-I = 2.95 Å, (C), Pt-Cl = 2.55 Å, (B) and Pt-O = 2.1 Å, (A)). However, the obtained Pt-I bond length value is higher than the experimental one reported for erythro-8-$PtI_2$ (2.566 Å, 2.574 Å) and erythro-9-$PtI_2$ (2.583 Å, 2.586 Å), (Table I) [18]. The calculated Pt-Cl bond length at the energy minimum is also higher than the value 2.31 Å in related compound meso-3-$PtCl_2$ [21].

The Pt-O bond length value is not known and comparison is not possible. The E vs. r(Pt-L) plots are drawn for a wide range of possible r values (1.4 - 3.8 Å). It is seen from the Fig. 4 that irrespective of the leaving group, the order of the energies at the minima is as follows:

| meso-1-$PtL_2$ | < | erythro-8-$PtL_2$ | < | erythro-9-$PtL_2$ | | |
|---|---|---|---|---|---|---|
| -1.22 | < | 4.16 | < | 6.78 | kcal $mol^{-1}$ | $PtI_2$ |
| 0.14 | < | 5.45 | < | 8.17 | kcal $mol^{-1}$ | $PtCl_2$ |
| 34.52 | < | 39.38 | < | 43.33 | kcal $mol^{-1}$ | $PtSO_4$ |

The same energy order was found for the entire range of studied r values although the energy differences become smaller when moving away from the minima.

From Fig. 4 one may conclude that the shape of all curves is almost independent of the type of the leaving group: the curves are shifted in E and r, but the curvature (a measure of the force constant for Pt-L) is almost the same (MM uses generic values for the three cases).

*Complexes with leaving group I.* The energy order, thus obtained, meso-1-$PtI_2$ < erythro-8-$PtI_2$ < erythro-9-$PtI_2$, is not exactly reverse to the known estrogen activity order [18], meso-1-$PtI_2$ > erythro-9-$PtI_2$ > erythro-8-$PtI_2$

The complex with the lowest energy (meso-1-$PtI_2$) has the highest activity. At the same time the energy order for the erythro species: erythro-8-$PtI_2$ < erythro-9-$PtI_2$ does not correlate reversibly with the known activity. However, this energy order was obtained with equal Pt-I bond distances for erythro-8-$PtI_2$ and erythro-9-$PtI_2$, namely Pt-I=2.670 Å (included in the database, see Appendix).

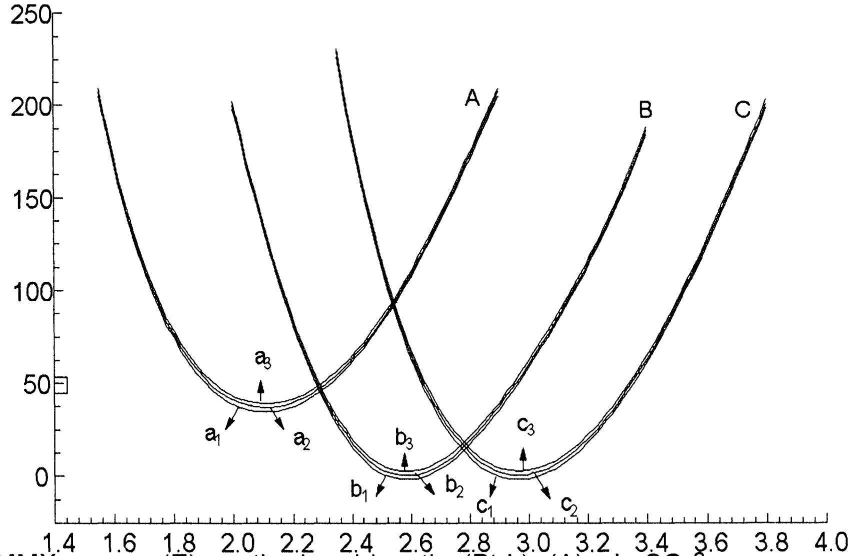

Fig. 4. The MMX energy (E) vs. the bond length r(Pt-L): (A) - L=$SO_4^{2-}$, $a_1$ - meso-1-$PtSO_4$, $a_2$ - erythro-8-$PtSO_4$, $a_3$ - erythro-9-$PtSO_4$, (B) - L=$Cl^-$; $b_1$ - meso-1-$PtCl_2$, $b_2$ - erythro-8-$PtCl_2$, $b_3$ - erythro-9-$PtCl_2$, (C) - L=$I^-$, $c_1$ - meso-1-$PtI_2$, $c_2$ - erythro-8-$PtI_2$, $c_3$ - erythro-9-$PtI_2$.

A survey of the X-ray data in Table I shows that erythro-8-$PtI_2$ has shorter Pt-I distances than those of erythro-9-$PtI_2$. Since the MM energy of erythro-8-$PtI_2$ is slightly lower in value as compared with that of erythro-9-$PtI_2$ we expected that the energy order erythro-8-$PtI_2$ < erythro-9-$PtI_2$ may be reversed when the real distances are taken into account. Thus, the geometry optimization of erythro-8-$PtI_2$ and erythro-9-$PtI_2$ was done with available X-ray data for Pt-N, Pt-I bond lengths and I-Pt-I, N-Pt-N bond angles (Table I) [18].

The other geometry parameters do not differ significantly from those included in the MM database (see Appendix). However, X-ray data are not available for the other complexes in this group: meso-1-$PtI_2$, erythro-5-$PtI_2$ and erythro-7-$PtI_2$. In these cases parameters close to those for erythro-9-$PtI_2$ were used in the geometry optimization. The reason to use these parameters is that the complexes with unknown structure have Cl substituents in 2 positions of the Ph rings as it is in erythro-9-$PtI_2$ (with the exception of erythro-5-$PtI_2$).

The complexes were modelled by constraining Pt in the plane of the ligand donor atoms. The calculated bond lengths and bond angles are compared with the experimental values for erythro-8-$PtI_2$ and erythro-9-$PtI_2$ in Table I. Within the constraints used in the calculations the experimental bond lengths and bond angles are reproduced quite well by MM calculations.

*Complexes with leaving group Cl.* The complexes with leaving group L = Cl were treated in the same way as those with L = I. Since X-ray diffraction data for this group of complexes are not available we used the Pt-Cl and Pt-N bond lengths and the Cl-Pt-Cl and N-Pt-N bond angles for meso-3-$PtCl_2$ (X = OH, Y = Z = H), namely r(Pt-Cl) = 2.31 Å, r(Pt-N) = 2.07 Å, <Cl-Pt-Cl = 92.4°, <N-Pt-N = 81.2° and <N-Pt-Cl = 93.3° [21,24]. The results thus obtained follow the trends already obtained for the $PtI_2$ complexes. The MMX energy order: meso-1-$PtCl_2$ < erythro-9-$PtCl_2$ < erythro-8-$PtCl_2$ (which correlates reversibly with the estrogen activity order) was obtained when shorter Pt-L bond lengths ($\Delta_{av} \cong 0.02$ Å) are assumed for the erythro-8-$PtCl_2$ as compared with the erythro-9-$PtCl_2$ (as for $PtI_2$ complexes). When optimization was carried out without shortening the Pt-L bond, the order: erythro-8-$PtCl_2$ < erythro-9-$PtCl_2$ was obtained. The meso-1-$PtCl_2$ has always the lowest energy.

Table I. MMX energies and structural parameters (calculated and experimental) for erythro-8-$PtI_2$ and erythro-9-$PtI_2$.

| Complex | erythro-8-$PtI_2$, δ conformer | | erythro-9-$PtI_2$, λ conformer | |
|---|---|---|---|---|
| | calc. (this work) | exp. [16] | calc. (this work) | exp.[16] |
| MMXE(kcal $mol^{-1}$) | 74.39 | | 71.82 | |
| r($Pt-I_1$) (ang) | 2.566 | 2.566(2) | 2.584 | 2.583(2) |
| r($Pt-I_2$) (ang) | 2.579 | 2.574(2) | 2.586 | 2.586(2) |
| r($Pt-N_1$) (ang) | 2.078 | 2.09(1) | 2.123 | 2.05(1) |
| r($Pt-N_2$) (ang) | 2.115 | 1.96(1) | 2.167 | 2.08(1) |
| $\angle(I_1-Pt-I_2)$ (deg) | 94.64 | 94.15(7) | 94.39 | 94.59(4) |
| $\angle(N_1-Pt-N_2)$ (deg) | 82.73 | 82.10(4) | 81.50 | 82.1(4) |
| $\angle(I_1-Pt-N_1)$ (deg) | 93.04 | 93.70(4) | 93.14 | 91.9(3) |
| $\angle(I_2-Pt-N_2)$ (deg) | 89.58 | 90.0(3) | 90.97 | 90.2(3) |
| $\angle(I_1-Pt-N_2)$ (deg) | 175.73 | 175.5(4) | 174.51 | 175.1(3) |
| $\angle(I_2-Pt-N_1)$ (deg) | 172.24 | 171.8(4) | 172.47 | 173.1(3) |
| $\angle(N_1-C_1-C_2-N_2)$ (deg) | 54.35 | 51.90 | -50.53 | -51.50 |
| $\angle(N_2-Pt-N_1-C_1)$ (deg) | 19.46 | 17.79 | -24.57 | -12.45 |
| $\angle(N_1-Pt-N_2-C_2)$ (deg) | 10.01 | 12.35 | -2.21 | 16.37 |
| $\angle(N_1-C_1-C_{11}-C_{12})$ (deg) | -146.62 | -141.70 | 145.19 | 150.04 |
| $\angle(N_2-C_2-C_{21}-C_{22})$ (deg) | 149.67 | 151.68 | 37.51 | 41.81 |
| d(O-O) (ang) | 8.40 | 8.10 | 8.27 | 7.80 |

*Complexes with leaving group $SO_4^{2-}$.* The complexes with L = $SO_4^{2-}$ were optimized in the same way. No X-ray diffraction data are available for this group of complexes and for this reason parameters from the MM2 database were used. The question whether water-containing (ethylenediamine) (sulfato)platinum(II) complexes exist as unidentate aqua (ethylenediamine) (sulfato)platinum(II) complexes (Fig. 5a) or as diaqua (ethylenediamine) platinum(II)sulfates (Fig. 5d) was decided in favour of the Rochon and Melanson structure (Fig. 5a) by X-ray diffraction [22]: in (N, N-dimethylenediamine) (sulfato) platinum(II) complexes with 2 $H_2O$ molecules, the sulfate ion is a unidentate ligand and another site in the coordination sphere of Pt is occupied by $H_2O$.

The antitumor activity of the (ethylenediamine) (sulfato)platinum(II) complexes is not affected by differences in structures (a) and (d), since in aqueous solutions the sulfato group from the unidentate structure (a) in (ethylenediamine)(sulfato)platinum(II) complexes is quickly replaced by $H_2O$ molecules, thus forming the active diaqua(ethylenediamine)platinum(II) ion (d) [22].

All complexes in this group were modelled as unidentate monoaqua (ethylenediamine) (sulfato)platinum(II) complexes ($L_1 = SO_4^{2-}$ and $L_2 = H_2O$).

The energy order obtained at the minima of the $\underline{E}$ vs.r($Pt-OSO_3$) correlation curves is:

meso-1-$PtSO_4$ < erythro-8-$PtSO_4$ < erythro-9-$PtSO_4$.

Unfortunately, X-ray data for Pt-O bond distances are not available and we do not know how far from the theoretical minimum ($r_{Pt-O} = 2.1$ ≈) are the experimental bond lengths. In order to obtain the energy order erythro-9-$PtSO_4$ < erythro-8-$PtSO_4$, a shorter (as compared with 2.1 ≈, obtained at the minimum) Pt-O bond distance ($r_{Pt-O} = 1.9$ ≈) in erythro-8-$PtSO_4$ was used.

**B. Influence of the type and the positions of the ring substituents on the calculated energies and the estrogen activities (Table II)**

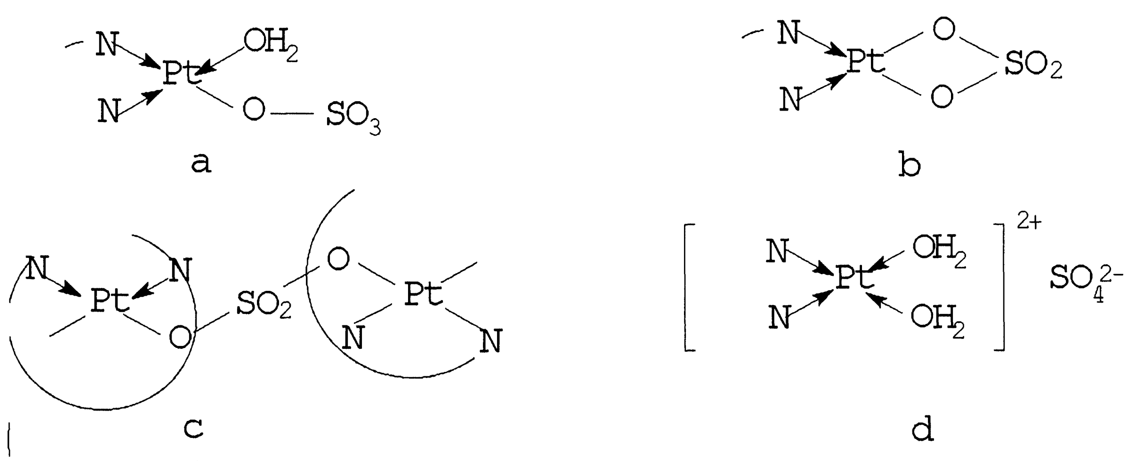

Fig. 5. Structures of water-containing (ethylenediamine)(sulfato)platinum(II) complexes: a - unidentate monoaqua(ethylenediamine)(sulfato)platinum(II), b,c - bidentate (ethylenediamine)(sulfato)platinum(II), d - unidentate diaqua(ethylenediamine)(sulfato)platinum(II) [12]

Table II. MMX energies (in kcal mol$^{-1}$) for meso-1-$PtL_2$, erythro-5-$PtL_2$, erythro-7-$PtL_2$, erythro-8-$PtL_2$ and erythro-9-$PtL_2$ (L = $I^-$, $Cl^-$, $SO_4^{2-}$).

| Complex | meso-λ | meso-δ | d,l-δ | d,l-λ |
|---|---|---|---|---|
| meso-1-$PtI_2$ | **67.56** | 68.40 | 71.05 | 65.87 |
| erythro-5-$PtI_2$ | 77.55 | 77.81 | 77.49 | 76.06 |
| erythro-7-$PtI_2$ | 70.08 | 70.27 | 73.74 | 68.26 |
| erythro-8-$PtI_2$ | 75.65 | **74.39** | 74.12 | 76.07 |
| erythro-9-$PtI_2$ | **71.82** | 72.39 | 73.99 | 71.77 |
| meso-1-$PtCl_2$ | **32.37** | 32.81 | 32.57 | 30.59 |
| erythro-5-$PtCl_2$ | 41.63 | 42.15 | 39.41 | 40.82 |
| erythro-7-$PtCl_2$ | 34.49 | 34.89 | 34.95 | 32.96 |
| erythro-8-$PtCl_2$ | 39.56 | **39.20** | 38.58 | 38.43 |
| erythro-9-$PtCl_2$ | **36.28** | 36.47 | 35.72 | 36.37 |
| meso-1-$PtSO_4$ | **34.52** | 34.81 | 36.64 | 30.68 |
| erythro-5-$PtSO_4$ | 44.09 | 44.41 | 41.06 | 40.74 |
| erythro-7-$PtSO_4$ | 36.84 | 37.02 | 38.19 | 32.76 |
| erythro-8-$PtSO_4$ | 40.94 | **41.17** | 40.32 | 37.07 |
| erythro-9-$PtSO_4$ | **39.04** | 38.90 | 39.69 | 36.46 |

Recently we have studied in details the influence of the type of the ring substituents (Cl, F and OH) and their positions in the phenyl rings (2, 3, 4, 5 and 6) on the calculated energies and thermodynamic stabilities of the studied compounds [26]. Here we will present only an essential part of the results.

*Complexes with leaving group I.* The calculated MMX energies for the complexes with leaving group I are given in Table II (see also [26]).

*Meso-1-PtI$_2$*. Meso-1-$PtI_2$ (four Cl substituents in 2- and 6-positions of the aromatic rings) was with the lowest energy (67.56 kcal mol$^{-1}$) in the studied series of meso isomers. This complex has the highest estrogen activity among all the tested compounds of this group [18]. The calculated energies for all the studied compounds follow the order:

meso-1-$PtI_2$ < erythro-7-$PtI_2$ < erythro-9-$PtI_2$ < erythro-8-$PtI_2$ < erythro-5-$PtI_2$.

*Erythro-9-PtI$_2$ and erythro-8-PtI$_2$*. Erythro-9-$PtI_2$ and erythro-8-$PtI_2$ have also shown estrogen properties and the estrogen activity order: meso-1-$PtI_2$ > erythro-9-$PtI_2$ > erythro-8-$PtI_2$ [18] follows the energy order we found after taking into account the real bond lengths: meso-1-$PtI_2$ < erythro-9-$PtI_2$ < erythro-8-$PtI_2$. As seen from Table II the calculated energy order is reversed with respect to the estrogen activity order; the complex with the lowest energy has the best estrogen activity (meso-1-$PtI_2$).

Erythro-8-$PtI_2$ and erythro-9-$PtI_2$ have different X substituents: for erythro-8-$PtI_2$, X = F and for erythro-9-$PtI_2$, X = Cl (Fig. 2). Four other complexes (obtained by exchanging the X and Y positions) were included in the calculations [26]. The calculated MMX energies for erythro-8-$PtI_2$ and erythro-9-$PtI_2$ are given in Table III.

The results in Table III show that for erythro-8-$PtI_2$ the lowest energy isomer is the δ-conformer, with X = F in 2-position of the axial aromatic ring (MMX = 74.39 kcal $mol^{-1}$, Fig. 2c). X-ray diffraction data reveal that the erythro-8-$PtI_2$ exists namely as meso isomer in δ conformation [18]. All other isomers are with higher energies. At the same time for erythro-9-$PtI_2$ the lowest energy conformer is the λ conformer, with X=Cl in 2-position of the axial aromatic ring (MMX=71.82 kcal $mol^{-1}$, Fig. 2b). This is again in full agreement with available X-ray and NMR data which show that erythro-9-$PtI_2$ exists as meso isomer in λ conformation. These two complexes, erythro-8-$PtI_2$ (δ-conformer) and erythro-9-$PtI_2$ (λ-conformer) were tested and they show antitumor activity against estrogen positive tumors [18]. If we compare the energies of these two complexes (74.39 and 71.82 kcal $mol^{-1}$) the "preferred" one is erythro-9-$PtI_2$ (erythro-9-$PtI_2$ has higher activity than erythro-8-$PtI_2$). They are both less active (higher energy) as compared with meso-1-$PtI_2$ (67.56 kcal $mol^{-1}$). Table III. MMX energies (in kcal $mol^{-1}$) for erythro-8-$PtL_2$ and erythro-9-$PtL_2$ (L = $I^-$, $Cl^-$, $SO_4^{2-}$) with different substituents.

| Complex | X and Y are in the equatorial ring | | X and Y are in the axial ring | |
|---|---|---|---|---|
| **erythro-8-$PtI_2$ meso- δ** | 78.122a | 79.49* | **74.39**2c | 77.89** |
| erythro-8-$PtI_2$ meso-λ | 78.872d | 78.25* | 75.652b | 78.78** |
| erythro-9-$PtI_2$ meso-δ | 76.242a | 76.99* | 72.392c | 77.46** |
| **erythro-9-$PtI_2$ meso-λ** | 74.932d | 75.10* | **71.82**2b | 76.07** |
| **erythro-8-$PtCl_2$ meso-δ** | 43.072a | 42.87* | **39.20**2c | 42.45** |
| erythro-8-$PtCl_2$ meso-λ | 42.192d | 42.01* | 39.562b | 42.79** |
| erythro-9-$PtCl_2$ meso-δ | 38.782a | 39.30* | 36.472c | 40.08** |
| **erythro-9-$PtCl_2$ meso-λ** | 39.532d | 39.58* | **36.29**2b | 40.17** |
| **erythro-8-$PtSO_4$ meso-δ** | 43.392a | 42.98* | 41.172c | 43.67** |
| erythro-8-$PtSO_4$ meso-λ | 43.932d | 43.79* | **40.952b** | 43.84** |
| erythro-9-$PtSO_4$ meso-δ | 41.692a | 41.77* | 38.902c | 43.51** |
| **erythro-9-$PtSO_4$ meso-λ** | 41.822d | 41.95* | **39.04**2b | 42.45** |

2a, 2d - energies of the 2a and 2d species (Fig.2a and Fig. 2d); X (Cl or F) is in 2-position;
* - energies of the 2a and 2d species obtained after exchange of the X and Y positions, X (Cl or F) is in 6-position;
2b, 2c - energies of the 2b and 2c species (Fig.2b and Fig. 2c); X (Cl or F) is in 2-position;
** - energies of the 2b and 2c species obtained after exchange of the X and Y positions, X (Cl or F) is in 6-position.

The results in Table III show also the following trends:

(i) when the X and Y substituents are in an equatorial aromatic ring (Fig. 2a and 2d) the exchange of their positions does not influence significantly the calculated MMX energies (compare first and second columns of Table III).

(ii) when the X and Y substituents are in the axial aromatic ring (Fig. 2b and 2c) the exchange of their positions increases the calculated energies (compare third and fourth columns of

Table III). Obviously, the "preferred" complexes are those with substituents in the axial ring and Cl (F) atom in 2-position of the aromatic ring. In the case of erythro-8-$PtI_2$ this is the δ-conformer and in the case of erythro-9-$PtI_2$ this is the λ-conformer (both were prepared also experimentally).

*Erythro-7-$PtI_2$.*. Erythro-7-$PtI_2$ has also a low energy (MMX =70.08 kcal $mol^{-1}$) as compared with meso-1-$PtI_2$ (see the first column of Table II). This complex, however, has no estrogen activity. This finding was explained by the absence of OH- substituent in para-position which determines the estrogen properties of the complex. It was accepted that the estrogen receptor of the tumor cell binds the complexes through H-bonds between two OHs in the aromatic rings and binding sites of the receptor [7].

*Erythro-5-$PI_2$.. Number of Cl atoms in the aromatic rings*. The low energy of erythro-7-$PtI_2$ (MMX = 70.08 kcal $mol^{-1}$) is due to the presence of four Cl atoms in the aromatic rings as in meso-1-$PtI_2$ (MMX = 67.56 kcal $mol^{-1}$). With three Cl atoms (erythro-9-$PtI_2$) the energy is higher (71.82 kcal $mol^{-1}$) and with two (erythro-5-$PI_2$) the energy has the highest value (MMX = 77.55 kcal $mol^{-1}$) (Table II). For the complexes with X = Cl the obtained estrogen activity was the highest one. This is in agreement with the assumption that the presence of Cl in the aromatic rings increases the lipophilic nature of the compounds improving their binding possibility [18].

*d,l-δ and d,l-λ conformers*. The energy order found for the d,l-δ and d,l-λ conformers (threo) is as follows:

D,L-1-$PtI_2$ < Threo-7-$PtI_2$ < Threo-9-$PtI_2$ < Threo-8-$PtI_2$ < Threo-5-$PtI_2$.

This order (third and fourth columns of Table II) is the same as for the meso-δ and meso-λ conformers of the studied complexes.

*Comparison of the conformational energies of the four isomers meso-δ, meso-λ, d,l-δ and d,l-λ) for every complex*. For meso-1-$PtI_2$ (D,L-1-$PtI_2$), erythro-7-$PtI_2$ (threo-7-$PtI_2$) and erythro-9-$PI_2$ (threo-9-$PtI_2$) the following order was found (1-, 3- and 5 rows in Table II):

d,l-λ < meso-λ ≅ meso-δ < d,l-δ.

For erythro-5-$PtI_2$ (threo-5-$PtI_2$) again the λ-conformers are preferred but the order of the δ-conformers is reverse (2 row in Table II):

d,l-λ < meso-λ ≅ d,l-δ ≅ meso-δ.

As seen from Table II the d,l-λ isomers have lower energies as compared with the meso-λ isomers. For erythro-9-$PtI_2$ an d,l-λ isomer has also been found but its estrogen activity was relatively low [7]. This was explained in terms of two factors: (a) the spatial location of the two N atoms, which was different from that in the meso isomers; (b) the lack of flexibility in the five-membered chelate ring which hinders the approach of the two Ph rings and O-O distance decrease as is the case in the meso series [7]. It is possible that these complexes, d,l-λ, have another mechanism of action (a hydrolysis mechanism). Recently, we have shown that the energy of d,l-3-$PtCl_2$, d,l-λ, is lower than the energy of the meso-3-$PtCl_2$, meso-λ, and the rate of hydrolysis and antitumor activity of the first compound are higher than those of meso-3-$PtCl_2$ [24]. On the basis of these results, reported elsewhere [24], the hydrolysis mechanism of action may be assumed for the d,l-λ isomer. Obviously, regardless of the mechanism of action, the complexes used as antitumor agents should be thermodynamically stable and should not undergo kinetic substitutions in order to reach the cell unchanged and to attack subsequently the critical area of DNA.

The only compound in this series that preferred the δ conformation is erythro-8-$PtI_2$:

meso-δ ≅ d,l-δ < meso-λ < d,l-λ.

The calculated energies are in agreement with the X-ray diffraction data, which reveal meso isomer in δ conformation for erythro-8-$PtI_2$ and meso isomer in λ conformation for erythro-9-$PtI_2$ [18].

*Complexes with leaving group $Cl^-$*. The calculated energies are given in Table II and they follow the order:

meso-1-$PtCl_2$ < erythro-7-$PtCl_2$ < erythro-9-$PtCl_2$ < erythro-8-$PtCl_2$ < erythro-5-$PtCl_2$.

For erythro-8-$PtI_2$ and erythro-9-$PtI_2$, four other complexes (obtained after exchanging the X and Y positions) were included in the calculations. The calculated MMX energies for all complexes of erythro-8-$PtI_2$ and erythro-9-$PtI_2$ are given in Table III.

The results for the complexes with leaving group Cl show that: (i) meso-λ and meso-δ conformers differ only slightly in energy; (ii) the d,l-λ isomers have always lower energies as compared with both meso isomers.

*Complexes with leaving group $SO_4^{2-}$*. The calculated energies for this group complexes follow the same order (Table II):

meso-1-$PtSO_4$ < erythro-7-$PtSO_4$ < erythro-9-$PtSO_4$ < erythro-8-$PtSO_4$ < erythro-5-$PtSO_4$.

The results for this group of complexes confirm the trends obtained for the other two groups (L = Cl and I): (i) the meso-1-$PtSO_4$ has always the lowest energy and erythro-9-$PtSO_4$ <

erythro-8-$PtSO_4$ for $r_{Pt-O}$ = 1.9 Å; (ii) the d,l-λ are preferred for all complexes; (iii). meso-λ and meso-δ conformers differ only slightly in energy. The results for erythro-8-$PtSO_4$ and erythro-9-$PtSO_4$ complexes are compared in Table III. The exchange of X and Y substituents in the axial ring increases the energy while the exchange of the substituent positions in the equatorial ring does not bring significant energy changes.

**C. Rotational barriers about $C_{sp3}$ -$C_{ar}$ bonds.**

In order to obtain the preferred orientations of the aromatic rings the rotational energies, $E_{rot}$, about $C_{sp3}$ - $C_{ar}$ bonds were calculated for all complexes starting from the optimized geometries and rotating from 0 to 360 deg, with 10 deg increment. The following trends were found:

(A) All complexes have their energy minimum at the starting (optimized) geometries and another minimum at 180 deg. The second minimum is higher in energy (by 2-10 kcal $mol^{-1}$) as compared with the energy of the optimized geometry ($E_{rot}$ = 0.0 kcal $mol^{-1}$). The rotational barriers are different for the complexes with substituted and unsubstituted (or partially substituted) aromatic ring:

1. For all meso and d,l-λ isomers a) The rotation about $C_{sp3}$ -$C_{ar}$ bonds of the substituted aromatic ring (with four Cl substituents in 2- and 6-positions of the aromatic rings (meso-1-$PtL_2$, erythro-7-$PtL_2$), is highly unfavorable since the rotational barriers are very high (>> 100 kcal $mol^{-1}$).

b) In the case of erythro-8-$PtI_2$ and erythro-9-$PtI_2$ (three Cl substituent in 2- or 6-positions) the barriers obtained are lower in energy (< 100 kcal $mol^{-1}$) but rotation is still unfavorable.

c) In the case of erythro-5-$PtL_2$ (unsubstituted aromatic ring) the rotational energy about the $C_{sp}$-$C_{ar}$ bond of the unsubstituted aromatic ring is ~ 80 kcal $mol^{-1}$, for d,l isomer and ~ 100 kcal $mol^{-1}$, for meso isomer.

2. For the d,l isomers in δ conformation

The d,l-δ conformers have two other minima (at ~90 deg and ~270 deg ); they are much higher in energy than the minimum at 180 deg (by 20-70 kcal $mol^{-1}$); these minima are shallow and obviously not populated. One exception is d,l-δ erythro-5-$PtI_2$ (threo-5-$PtI_2$) where the three minima for the rotation energy of the unsubstituted aromatic ring have low values (6.30, 0.51 and 4.45 kcal $mol^{-1}$). This exception was explain in terms of the lack of substituents in one aromatic ring (X = Y = H). The calculated rotational barriers (~9 kcal $mol^{-1}$) are lower as compared with the barriers of the substituted ring and rotation about $C_{sp3}$ -$C_{ar}$ bond could be possible.

(B) The 2-position of Cl or F in the aromatic ring is preferred ($E_{rot}$ = -3.00 kcal $mol^{-1}$) as compared with the 6-position ($E_{rot}$ = 0.00 kcal $mol^{-1}$).

### Conclusions

The results obtained in this work show that:

(a) - The MM calculations predict correctly the absolute conformation as found in the solid state by X-ray diffraction, and hence they can be expected to predict correctly the conformations for which X-ray diffraction data are not available;

(b) - The calculated energies and the stabilities of the studied complexes (meso-1-$PtL_2$, erythro-9-$PtL_2$ and erythro-8-$PtL_2$) on one side and their estrogen affinity and activity on the other side are found to run parallel: the most stable complex has the highest estrogen activity. Such trends may help further the selection of new complexes to test for their biological properties.

(c) - The type and the positions of the ring substituents influence the calculated energies. When the X and Y substituents are in an equatorial aromatic ring, the exchange of their positions does not influence the calculated MMX energy. Conversely, when the X and Y substituents are in the axial aromatic ring, the exchange of their positions increases the calculated energies. The "preferred" complexes and conformations are those with axial ring substituents and Cl (or F) atoms in 2-position of the aromatic ring: the δ-conformer for erythro-8-$PtI_2$ and λ-conformer for erythro-9-$PtI_2$. Both compounds were prepared also experimentally in these conformations.

(d) - The type of the leaving group is of minor importance for the calculated energy order and estrogen activity;

(e) - The calculated rotational barriers about the $C_{sp3}$-$C_{sp2}$ bonds when both aromatic rings are substituted (meso-1-$PtL_2$, erythro-7-$PtL_2$, erythro-8-$PtL_2$, erythro-9-$PtL_2$) are very high and rotations about these bonds are unfavorable. In the case of erythro-5-$PtL_2$ (one ring is unsubstituted) rotation about the $C_{sp3}$-$C_{sp2}$ bond of the unsubstituted aromatic ring is probable since the rotational barrier is low (~ 9 kcal $mol^{-1}$).

### Acknowledgments

This work was supported by the Bulgarian National Research Fund through Grant X-647. The authors thank Prof. H. Schönenberger and Prof. R. Gust for the experimental data cited in this paper.

**References**

1. B. Rosenberg, L. van Camp, J. E. Trosko, V. H. Mansour, *Nature,* **222** (1969) 385.
2. I. H. Krakoff, *Platinum and Other Metal Coordination Compounds in Cancer Chemotherapy: Clinical Applications of Platinum Complexes* (Nicolini M, ed) Martinus Nijhoff Publishing, Boston, 351 (1988).
3. P. J. Löher Sr, L. H. Einhorn, *Ann Intern Med.*, **100** (1984) 704.
4. P. J. Löher Sr, S. D. Williams, L. H. Einhorn, *J. Natl. Cancer. Inst.,* **80** (1988) 1373.
5. R. Gust and H. Schönenberger, *Eur. J. Med. Chem.,* **28** (1993) 103.
6. P. J. Bednarski, R. Gust, T. Spruss, N. Knebel, A. Otto, M. Farbel, R. Koop, E. Holler, Ervin von Angerer and H. Schönenberger, *Cancer Treatment Revs,* **17** (1990) 221.
7. R. Gust, K. Niebler and H. Schönenberger, im press ( *J. Med. Chem.)*
8. R. Gust, H. Schönenberger, Klaus-Jörgen Range, U. Klement and M. Schneider, *Monatshefte für Chemie,* **124** (1993) 1181.
9. T. Spruss, S. Scherte, M. Schneider, R. Gust, K. Bauer and H. Schönenberger, *J. Cancer. Res. Clin. Oncol.,* **119** (1993) 707.
10. M. Jennerwein, B. Wappes, R. Gust, H. Schönenberger, J. Engel, S. Seeber, R. Osieka, *J. Cancer Res. Clin. Oncol.,* **114** (1988) 347.
11. M. Jennerwein, R. Gust, R. Müller, H. Schönenberger, J. Engel, M. R. Berger, D. Schm-hl, S. Seeber, R. Osieka, G. Atassi, D. M.-De Bock, *Arch. Pharm.,* **322** (1989) 67.
12. R. Gust, T. Burgemeister, A. Mannschreck and H. Schönenberger, *J. Med. Chem.,* **33** (1990) 2535.
13. R. Müller, R. Gust, G. Bernhardt, C. Keller, H. Schänenberger, S. Seeber, R. Osieka, A. Eastman, M. Jennerwein, *J. Cancer Res. Clin. Oncol.,* **116** (1990) 237.
14. R. Gust and H. Schönenberger, *Arch. Pharm.* (Weinheim), **327** (1994) 763.
15. J. Karl, R.Gust, T. Spruss, M. Schneider, H. Schönenberger, J. Engel, Karl- Heinz Wrobel, F. Lux and S. T. Haeberlin, *J. Med. Chem.,* **31** (1988) 72.
16. J. A. Katzenellenbogen and B. S. Katzenellenbogen, *Breast Cancer Res. Treat.,* **2** (1982) 347.
17. B. Wappes, M. Jennerwein, E. von Angerer, H. Schönenberger, J. Engel, M. R. Berger and K.-H. Wrobel, *J. Med. Chem.,,* **27** (1984) 1280.
18. R. Gust, H. Schönenberger, U. Klement and Klaus-Jörgen Range, *Arch. Pharm. (Weinheim),* **326** (1993) 967.
19. R. Gust and H. Schönenberger, *Arch. Pharm. (Weinheim),* **326** (1993) 405.
20. T. Spruss, R.Gust, R. M+ller, J. Engel, and H. Schönenberger, *Arch. Pharm. (Weinheim,)* **323** (1990) 99.
21. R. Gust, H. Schönenberger, J. Kritzenberger, K.-J. Range, U. Klementm T. Burgemeister, *Inorg. Chem.,* **32** (1993) 5939.
22. F. D. Rochow and R. Melanson, *Inorg. Chem.,* **26** (1987) 989.
23. S. Shertl, R. Gust, R. Müller, T. Spruss and H. Schönenberger, *Arch. Pharm. (Weinheim),* **325** (1992) 113.
24. G. St. Nikolov, N. Trendafilova, H. Schönenberger, R.Gust, J. Kritzenberger and H. Yersin, *Inorg. Chim. Acta,* **217** (1994) 159.
25. G.St. Nikolov, N. Trendafilova, I. Georgieva, H. Schönenberger, R. Gust, J. Kritzenberger and H. Yersin, *Monatshefte für Chemie,* **128** (1997) 443.
26. I. Georgieva and N. Trendafilova, *Monatshefte für Chemie,* **128** (1997) 1119.
27. M. Zimmer, *Chem. Rev.*, **95 (**1995) 2629.
28. L. J. DeHayes and D. H. Busch, *Inorg. Chem.,* **12** (1973) 1505 and refs therein.
29. J. R. Gollogly and Hawkins, *Inorg. Chem.*, **8** (1969) 1168.
30. J. R. Gollogly and Hawkins, *Inorg. Chem.,* **11** (1972) 156.
31. Y. Yoshikawa, *J. Comput. Chem.*, **11** (1990) 326.
32. P. Comba, *Coord. Chem. Revs.*, **123** (1993) 1 and refs therein.
33. P. Comba, M. Zimmer, *Inorg. Chem.*, **33** (1994) 5368.
34. P. V. Bernhardt and P. Comba, *Inorg. Chem.*, **31** (1992) 2638.
35. P. Comba, *Comments on Inorg. Chem..*, **16** (1994) 3.
36. H. Basch, M. Krauss, W. J. Stevens and D. Cohen, *Inorg. Chem.*, **24** (1985) 3313.
37. a) T. W. Hambley, *Inorg. Chem.*, **30,** 937 (1991); b) T. W. Hambley, *Inorg. Chem.,* **27** (1988) 1073; c) T. W. Hambley, C. J. Hawkins, J. A. Palmer, M. R. Snow, *Aust. J. Chem.*, **34** (1981) 45; d) M. R. Snow, *J.Am. Chem. Soc.,* **92** (1970) 3610; e) D. A. Buckingham, I. E. Maxwell, M. R. Snow, *J.Am. Chem. Soc.,* **92** (1970) 3617.
38. MMX, *Q.C.P.E.* 395, Bloomington, IN, USA.
39. Clark, *A Handbook of Computational Chemistry* J. Wiley 1985.

### Appendix

The MMX force field includes the folowing terms: bond stretch, $E_r$, valence angle deformation, $E_v$, cross-term for bond-angle interaction, $E_{r-v}$, torsional energy, $E_t$, van der Waals (non-bonded) interactions, $E_{vdw}$, dipole-dipole interaction, $E_{dd}$.. Both $E_r$ and $E_v$ are treated by the Hook law. Below are given the set of parameters used in this work.

| Stretch | $k_r$ (mdyn Å$^{-1}$) | | Standard bond length (Å) | | bond moment (D) |
|---|---|---|---|---|---|
| | this work | literature | this work | literature | |
| Pt - LP | 2.000 | - | 0.800 | - | - |
| Pt - Cl | 2.000 | 1.47[28] | 2.300 | 2.30[28] | 0.000 |
| Pt - N | 2.000 | 2.54[37b]<br>1.68[28] | 1.980 | 2.03[37b]<br>2.00[28] | 0.000 |
| Pt - O | 2.000 | - | 1.800 | - | 0.000 |
| Pt - O$^-$ | 2.000 | - | 1.800 | - | - |
| N - C | 5.100 | 6.00[37c, 34] | 1.440 | 1.49[37c, 34] | 0.040 |
| C - C | 4.400 | 5.00[37c, 34]<br>4.50[28] | 1.523 | 1.50[37c, 34]<br>1.54[28] | 0.000 |
| N - H | 6.100 | 5.64[37c, 34]<br>4.92[28] | 1.015 | 0.91[37c, 34]<br>1.00[28] | -0.760 |
| C - H | 4.600 | 5.00[37c, 34]<br>4.55[28] | 1.113 | 0.97[37c, 34]<br>1.09[28] | 0.000 |
| C - $C_{ar}$ | 5.000 | 5.00[34] | 1.497 | 1.50[34] | 0.000 |
| $C_{ar}$ - $C_{ar}$ | 9.600 | - | 1.337 | - | 0.000 |
| $C_{ar}$ - H | 4.600 | 5.00[34] | 1.101 | 0.97[34] | -0.200 |
| O - H | 4.600 | 5.00[34] | 0.942 | 0.91[34] | -1.115 |
| $C_{ar}$ - O(H) | 6.800 | - | 1.355 | - | 0.000 |
| N - LP | 4.500 | - | 0.600 | - | 0.600 |
| O - LP | 4.500 | - | 0.600 | - | 0.900 |

| Bend | $k_\theta$ (mdyn Å$^{-1}$) | | Standard Valence angle (deg.) | |
|---|---|---|---|---|
| | this work | literature | this work | literature |
| Cl - Pt - Cl | 0.000 | 0.000[34], 0.28[29,30,37] | 90, 120, 180 | 86.6[29,30,37] |
| Cl - Pt - N | 0.000 | 0.000[34], 0.28[29,30,37] | 90, 120, 180 | 86.6[29,30,37] |
| Cl - Pt - O | 0.000 | 0.000[34] | 90, 120, 180 | - |
| N - Pt - O | 0.000 | 0.000[34] | 90, 120, 180 | - |
| N - Pt - N | 0.000 | 0.000[34], 0.400[29,30,37] | 90, 120, 180 | 90.0[29,30,37] |
| Pt - N - C | 0.000 | 0.300[37b], 0.280[29,30,37]<br>0.200[34] | | 127.3[37b], 110.0[34]<br>109.5[29,30,37] |
| Pt - O - H | 0.000 | 0.100[34] | | 109.7[34] |
| N - C - C | 0.570 | 0.500[37c], 0.45[34] | 109.47 | - |
| N - C - $C_{ar}$ | 1.045 | - | 110.74 | - |
| C - C - $C_{ar}$ | 0.450 | - | 109.47 | - |
| C - $C_{ar}$ - $C_{ar}$ | 0.550 | - | 121.40 | - |
| $C_{ar}$ - $C_{ar}$ - O | 0.700 | - | 124.30 | - |
| $C_{ar}$ -$C_{ar}$ -$C_{ar}$ | 0.430 | - | 120.00 | - |
| Pt - N - H | 0.000 | 0.100[34], 0.28[29,30,37] | 109.70 | 109.7[34],109.5[29,30,37] |
| Pt - N - LP | 0.000 | - | | - |
| Pt - O - LP | 0.000 | - | | - |
| H - N - H | 0.500 | 0.320[37c], 0.330[34] | 104.50 | 108.98[34] |
| H - N - C | 0.500 | 0.360[37c], 0.450[34] | 109.47 | 109.49[34] |
| LP - N - C | 0.500 | - | 109.20 | - |
| N - C - H | 0.500 | 0.360[37c] | 108.80 | 108.98[37c] |
| H - C - H | 0.320 | 0.320[37c] | 109.40 | 108.98[37c] |

| | | | | |
|---|---|---|---|---|
| C - C - H | 0.360 | 0.360[37c, 34]<br>0.450[34] | 109.39 | 109.38[37c] |
| $C_{ar}$ - O - LP | 0.100 | - | 120.00 | - |
| LP - O - LP | 0.240 | - | 131.00 | - |
| LP - O - H | 0.240 | - | 101.01 | - |
| LP - N - H | 0.500 | - | 108.00 | - |

| Torsional (this work) | $V_1$ | $V_2$ | $V_3$ |
|---|---|---|---|
| N - C - C - N | -0.400 | -1.100 | 1.200 |
| N - C - $C_{ar}$ - $C_{ar}$ | 0.000 | 0.000 | 0.000 |
| C - C - N - LP | 0.200 | -0.220 | 0.100 |
| $C_{ar}$ - C - N - LP | 0.000 | 0.000 | 0.000 |

| Van der Waals | R | | EPS | | LPD | IHTYP |
|---|---|---|---|---|---|---|
| | this work | literature | this work | literature | | |
| C (sp[3]) | 1.900 | 1.900[34] | 0.044 | 0.044[34] | 0 | 0 |
| C (sp[2]) | 1.940 | - | 0.044 | - | 110 | 0 |
| H (C - H) | 1.500 | 1.440[34] | 0.047 | 0.024[34] | 0 | 20 |
| O (C - OH) | 1.740 | 1.700[34] | 0.050 | 0.055[34] | 90 | 0 |
| N (sp[3]) | 1.820 | 1.800[34] | 0.055 | 0.050[34] | 200 | 0 |
| Cl | 2.030 | - | 0.240 | - | 10 | 0 |
| LP | 1.200 | - | 0.016 | - | 0 | 0 |
| $C_{ar}$ | 1.900 | - | 0.044 | - | 110 | 0 |
| H (OH) | 1.100 | - | 0.036 | - | 0 | 40 |
| H (NH) | 1.125 | - | 0.034 | - | 0 | 0 |
| M1 | 0.000 | - | 0.400 | - | 0 | 0 |
| M2 | 0.000 | - | 0.400 | - | 0 | 0 |
| M3 | 0.000 | - | 0.400 | - | 0 | 0 |
| spherical $H_2O$ | 1.530 | - | 0.500 | - | 0 | 0 |

| Out-of-plane bending (this work) | |
|---|---|
| C - $C_{ar}$ | 0.800 |
| $C_{ar}$ - $C_{ar}$ | 0.800 |
| C - O | 0.800 |

| Stretch - Bend (this work) | |
|---|---|
| Str.-bend (1) | 0.12 |
| Str.-bend (2) | 0.25 |
| Str.-bend (3) | 0.09 |
| Str.-bend (4) | -0.40 |

For explanation of the abbreviations used in this Table see [39].

**Received: January 5, 1998 - Accepted: February 13, 1998 - Received in revised camera-ready format: February 16, 1998**

